# Linking science and policy to support the implementation of the Minamata Convention on Mercury

**DOI:** 10.1007/s13280-017-1003-x

**Published:** 2018-01-31

**Authors:** Henrik Selin, Susan Egan Keane, Shuxiao Wang, Noelle E. Selin, Kenneth Davis, Dominique Bally

**Affiliations:** 1 Frederick S Pardee School of Global Studies, Boston University, 154 Bay State Road, Boston, MA 02215 USA; 2 Natural Resources Defense Council, 1152 15th St, NW, Suite 300, Washington, DC 20005 USA; 3 School of Environment, Tsinghua University, Beijing, 100084 China; 4 Institute for Data, Systems, and Society, and Department of Earth, Atmospheric, and Planetary Sciences, Massachusetts Institute of Technology, 77 Massachusetts Avenue, Cambridge, MA 02139 USA; 5 United Nations Environment Programme, Chemicals and Health Branch, International Environment House I, 11-13 chemin des Anemones, 1219 Geneva, Switzerland; 6 African Center for Environmental Health, BP 826, Cidex 03 Abidjan, Côte d’Ivoire

**Keywords:** Environmental treaty implementation, Mercury, Minamata Convention, Science–policy, Toxic pollution

## Abstract

The Minamata Convention on Mercury, with its objective to protect human health and the environment from the dangers of mercury (Hg), entered into force in 2017. The Convention outlines a life-cycle approach to the production, use, emissions, releases, handling, and disposal of Hg. As it moves into the implementation phase, scientific work and information are critically needed to support decision-making and management. This paper synthesizes existing knowledge and examines three areas in which researchers across the natural sciences, engineering, and social sciences can mobilize and disseminate knowledge in support of Hg abatement and the realization of the Convention’s objective: (1) uses, emissions, and releases; (2) support, awareness raising, and education; and (3) impacts and effectiveness. The paper ends with a discussion of the future of Hg science and policy.

## Introduction

**Fig. 1:**
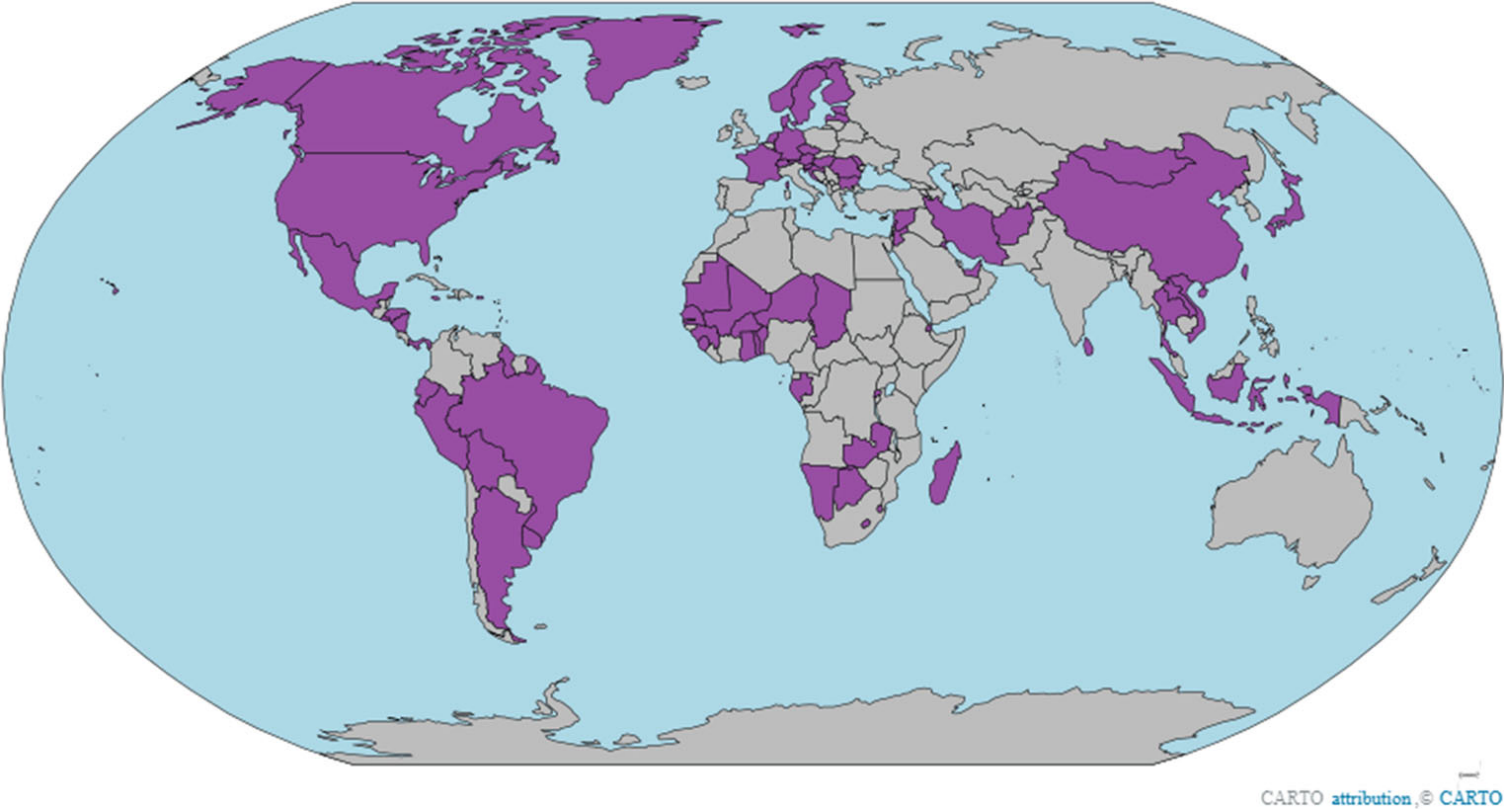
Map of convention parties

The Minamata Convention on Mercury, which aims to “protect human health and the environment from anthropogenic emissions and releases of mercury and mercury compounds” (Article 1), was adopted in 2013. The world’s countries, with the participation of many intergovernmental and non-governmental organizations, negotiated the Convention to outline a set of shared principles, standards, and rules (Andresen et al. 2013; Eriksen and Perrez 2014; Selin 2014a; You 2015). Countries voluntarily decide whether to become a party to the Convention, but once they commit to do so, its provisions are legally binding to all parties. The same is true for the European Union, which can join separately from its member states as a Regional Economic Integration Organization (Selin and VanDeveer 2015). The Convention entered into force on August 16, 2017, 90 days after it received its 50th ratification. As of November 2017, the Convention had 84 parties, and more countries are expected to join in the future (see Fig. 1).

The Minamata Convention builds upon an extensive body of scientific knowledge on mercury (Hg) and decades of policy efforts to manage its hazards. Science played a key role in establishing recognition for Hg as a global pollutant (United Nations Environment Programme 2002, 2008). Science has also helped reveal Hg’s global distribution (see companion paper by Obrist et al. 2018), its changing cycling in response to local perturbations (see companion paper by Hsu-Kim et al. 2018), and its health and environmental impacts (see companion paper by Eagles-Smith et al. 2018). Countries have worked together to address Hg risks since the 1970s (Selin and Selin 2006). Collaborative voluntary efforts under the United Nations Environment Programme (UNEP) Global Mercury Partnership to reduce Hg use and discharges and to draw attention to environmental and human health risks from Hg date back to the early 2000s (Sun 2017).

As the Minamata Convention moves into its implementation phase, different types of scientific research and information can support the Convention’s life-cycle approach to addressing the production, use, emissions, releases, handling, and disposal of Hg. The relationship between scientific information and environmental treaty implementation is complex, however. There is not a direct, causal one-way link between the generation of more scientific data and “better” policy-making and outcomes (Shackley and Wynne 1995). Rather, the linkages between the production of scientific information and policy-making are multifaceted, and the scope and character of connections between science and policy often vary across different environmental topics and venues. In addition, decision-makers will consider old and new scientific information in a context of broader (and sometimes competing) legal, political, economic, social, and cultural concerns and interests.

When examining science and policy linkages, it is necessary to pay attention both to the content of the scientific information and to the processes by which that information is produced and communicated. Research has shown that efforts to mobilize science to inform decision-making are most effective when the information is deemed scientifically credible, policy relevant, and politically legitimate by the intended users (Cash et al. 2003). To users of scientific information on Hg, scientific credibility is achieved by drawing upon well-established, peer-reviewed science. Although scientific credibility is essential, it alone is not enough—scientific information will more likely influence decision-making and management on Hg if it is also salient to the specific needs of policy-makers who are looking for policy relevant information. Political legitimacy refers to the perception of users that the scientific information on Hg is fair, unbiased, and respectful of different perspectives, values, and beliefs.

The purpose of this synthesis paper is to identify and discuss areas where researchers across the natural sciences, engineering, and social sciences can support the implementation of the Minamata Convention. In the following section, we provide a brief summary of the structure and content of Hg controls and management under the Convention. After that, we focus on how researchers can contribute in three broad Convention areas: (1) uses, emissions, and releases; (2) support, awareness raising, and education; and (3) impacts and effectiveness. In each area, we first synthesize the existing scientific knowledge base. We then discuss how different kinds of additional, scientifically credible information can aid Convention implementation, as well as some of the ways in which scientists can enhance the policy relevance and political legitimacy of such information. We end the paper with a discussion of major issues related to the future of Hg science and governance.

## Minamata Convention approaches to Hg management

Hg is a chemical element that is intentionally mined, used in products and industrial processes, and emitted and released into the environment as a by-product of human activities. The negotiators of the Minamata Convention designed an agreement with a life-cycle approach to cover all of these areas. They also needed to accommodate different national interests and find ways to assist developing countries with domestic implementation. While, as noted above, all parts of the Convention are legally binding for parties, some provisions require action (using the word “shall”) while some are hortatory (using words such as “should” or “may”). The core of the Convention is its control provisions and enabling provisions, which are described below. Other Convention Articles cover introductory material, definitions, and administrative matters. Table 1 lists key Convention dates and deadlines.

**Table 1:** Main Minamata Convention dates, requirements, and deadlines

UNEP Governing Council agrees to begin negotiations on a legally binding agreement on Hg	2009
Minamata Convention adopted and opened for signature	2013
Entry into force of the Minamata Convention	2017
First COP	2017
Prohibition of new Hg mining	Upon entry into force for a party
Phase-out of Hg use in acetaldehyde production	2018 (extension up to 10 years possible in some cases)
Deadline to reduce Hg use in VCM production by 50% (2010 baseline)	2020
Phase-out of Hg use in Hg-added products listed in Annex A to the Minamata Convention	2020 (extension up to 10 years possible in some cases)
Deadline for submitting ASGM National Action Plans to the Secretariat	3 years after entry into force for party (e.g., earliest 2020), or 3 years after notifying the Secretariat that ASGM activity is more than insignificant, whichever is later
Deadline for parties to require use of BAT and BEP for new sources from emissions categories listed in Annex D to the Minamata Convention	5 years after entry into force for a party (e.g., earliest 2022)
Start date for the COP to begin first effectiveness evaluations	No later than 2023
Phase-out of Hg use in chlor-alkali production	2025 (extension up to 10 years possible in some cases)
Deadline for parties to require use of ELV, BAT, BEP, or alternative measures for existing sources from emissions categories listed in Annex D to the Minamata Convention	10 years after entry into force for a party (e.g., earliest 2027)
Phase-out of existing primary Hg mining	15 years after entry into force for a party (e.g., earliest 2032)

*ASGM* artisanal and small-scale gold mining, *BAT* best available technique, *BEP* best environmental practice, *COP* conference of parties, *ELV* emission limit values, *Hg* mercury, *UNEP* United Nations Environment Programme, *VCM* vinyl chloride monomer

### Control provisions

The control provisions of the Minamata Convention (Articles 3–12) identify actions that the parties must take to address Hg supply, trade, use, emissions, and releases, and manage Hg wastes and Hg-contaminated sites. Article 3 covers the control of commercial Hg supply, including requiring the phase-out of production from primary mercury mining as well as banning the re-use of excess mercury from the decommissioning of chlor-alkali facilities—a leading industrial source of mercury supply. It also introduces trade restrictions for the export and import of Hg. These are accomplished through a system of prior informed consent whereby the national government of a domestic importer looking to buy Hg from a foreign source must officially give its written approval before the Hg can be legally imported from the foreign supplier, and the national government of the supplier must seek such formal prior approval on its behalf.

Articles 4–6 address Hg uses in products and processes. Article 4 prohibits the manufacture, import, and export of many Hg-added products, including certain types of batteries, switches, relays, lamps, pesticides, and cosmetics. Parties must take measures aimed at phasing down the use of dental fillings containing Hg amalgam, although no deadline is set. Article 5 obliges the parties to phase out Hg use in two manufacturing processes—chlor-alkali production and acetaldehyde production—and to restrict Hg use in three others—vinyl chloride monomer (VCM) production, sodium or potassium methylate or ethylate production, and polyurethane production. Article 6 allows parties to apply for time-limited extensions to phase out dates for Hg use in products and industrial processes covered by the Minamata Convention.

The Minamata Convention addresses Hg use in artisanal and small-scale gold mining (ASGM) in Article 7. This Article obligates parties to take steps to reduce and, where feasible, eliminate Hg use in, and emissions and releases from, ASGM. Parties with more than insignificant ASGM activities must develop and implement National Action Plans as part of their domestic efforts to address Hg-related problems and risks in this sector. Such actions are needed due to the fact that ASGM is the largest global source of Hg emissions and releases (United Nations Environment Programme 2013b), as ASGM is common in many developing countries where miners frequently operate in the informal sector. ASGM is also a major source of human exposure to inorganic Hg in many mining communities.

For Hg emissions to air and releases to land and water, treated separately in Articles 8 and 9, parties shall take measures to control and, where feasible, reduce emissions and releases. Parties shall use best available techniques (BATs), best environmental practices (BEPs), or emission limit values (ELVs) that are consistent with the application of BATs on new stationary sources of air emissions, including any new coal-fired power plants, coal-fired industrial boilers, non-ferrous metals processing, waste incineration, and cement clinker production. They can apply the same measures to such existing sources, but parties may also adopt alternative measures including a multi-pollutant strategy in which the control of Hg emissions is a co-benefit of methods applied to reduce other pollutants. Controls on Hg releases to water and land are implicitly incorporated into obligations for products, industrial processes, and sources of air emissions, but parties shall identify any additional relevant point sources to control releases from such sources.

Articles 10 and 11 cover Hg storage requirements and environmentally sound waste management practices. Parties must manage and dispose of discarded Hg and Hg-containing waste in an environmentally sound manner. Article 12 requires parties to endeavor to develop strategies for identifying and assessing Hg-contaminated sites, and also requires the Conference of the Parties (COP) to the Minamata Convention to develop guidance for the management of such sites.

### Enabling provisions

The enabling provisions of the Minamata Convention (Articles 13–24) are intended to help the parties implement and further develop the Convention, and track progress and measure the effectiveness of related management and policy measures. The collective application of these provisions is important to achieve effective treaty implementation among all parties, and to enhance the ability of different countries and stakeholders to generate scientifically credible information that is both salient to policy-making and viewed as politically legitimate.

To support and oversee treaty implementation, the Minamata Convention establishes an administrative Secretariat and the COP as the main decision-making body. The Convention also sets up a facilitative committee, an institutional mechanism, to promote implementation, review compliance, and explore ways to assist parties that encounter difficulties fulfilling their obligations. In developing countries, the awareness of the environmental and human health impacts of Hg and the availability of human, economic, scientific, and technical resources for comprehensive Hg management often are limited at national and local levels. Importantly, Article 13 defines a new mechanism for the provision of adequate, predictable, and timely financial resources to developing countries, which includes the Global Environment Facility Trust Fund and other funding sources. Further, Article 14 calls on parties to cooperate to provide capacity-building, technical assistance, and technology transfer to developing countries.

Article 16 encourages parties to promote the development and implementation of strategies to identify populations at risk from Hg exposure, to develop science-based public educational programs, to adopt science-based health guidelines on Hg exposure, and to strengthen health-care services to address Hg exposure. Article 17 stipulates that parties shall facilitate the exchange of scientific, technical, economic, and legal information. This includes information on the reduction or elimination of the production, use, trade, emissions, and releases of Hg; information about technically and economically viable alternatives for Hg use in products and processes; and epidemiological information concerning Hg-related health impacts. Under Article 18, parties shall promote and facilitate public information, awareness, and education about Hg.

Several Minamata Convention Articles are related to efforts to evaluate its effectiveness. Article 19 mandates that parties develop and improve methods for both modeling and monitoring of Hg in vulnerable human populations and in targeted environmental media. Article 20 stipulates that parties may develop and execute an implementation plan following an initial assessment, while Article 21 mandates that parties report on national measures and their effectiveness to the Secretariat. Article 15 creates the implementation and compliance committee to review progress and help parties that face challenges. Article 22 requires the COP to carry out periodic effectiveness evaluations using monitoring data combined with other scientific, environmental, technical, financial, and economic factors. The first of these must begin no later than 6 years after the Convention entered into force (2023 at the latest).

## Linking science and policy to implement the Minamata Convention

The scientific community can contribute to the implementation of the Minamata Convention for nearly all of the control and enabling provisions. Below, we group our discussion into three Convention areas: (1) uses, emissions, and releases; (2) support, awareness raising, and education; and (3) impacts and effectiveness. Table 2 shows the relationship of these areas with the relevant Convention Articles, and provides illustrative examples of potential scientific contributions. In each of the three Convention areas, we begin by synthesizing existing scientific knowledge. We then identify and discuss major knowledge gaps where scientists can generate additional credible information. We also suggest some ways that scientists can enhance the policy relevance and political legitimacy of such information.

**Table 2:** Three key convention areas and related articles

Area	Convention articles	Illustrative research needs
Uses, emissions, and releases	Article 3—Supply and trade Article 4—Products Article 5—Processes Article 6—Exemption to phase out dates Article 7—ASGM Article 8—Emissions to air Article 9—Releases to land or water Article 10—Storage Article 11—Waste Article 12—Contaminated sites	Evaluate availability and efficacy of Hg-free alternatives under a wide range of circumstances Improve methods for creating more reliable inventories of sources and emissions/releases Evaluate the effectiveness of control technologies Assist in the definition of BAT, BEP, and ELVs Support development of guidance on identification, characterization, risk assessment, and management of contaminated sites Quantify how mercury moves among land, water, and air to develop better controls
Support, awareness raising, and education	Article 13—Financial mechanism Article 14—Capacity-building, technical assistance, and technology transfer Article 16—Health Article 17—Information exchange Article 18—Public information, awareness, and education	Design and evaluate communication programs for education, training, and public awareness on Hg that respond to local conditions Assess risks and identify sources of exposure to vulnerable populations Engage in information exchange on technically and economically viable alternatives to Hg use Support technology transfer that addresses local social drivers of existing Hg use
Impacts and effectiveness	Article 15—Implementation and compliance Article 19—Research, development, and monitoring Article 20—Implementation plans Article 21—Reporting Article 22—Effectiveness evaluation	Expand tools and networks for Hg monitoring Provide baseline data and ensure comparability of Hg measurements Improve modeling methods to evaluate impacts from changes in emissions and releases Develop methods for collecting and compiling implementation, compliance, and reporting data Develop methods to integrate varied data into an effectiveness evaluation framework

### Uses, emissions, and releases

This area comprises the control provisions of the Minamata Convention. The comprehensive implementation of those Articles is essential to meet the Convention’s environmental and human health objective.

#### Synthesis of existing knowledge base

**Fig. 2:**
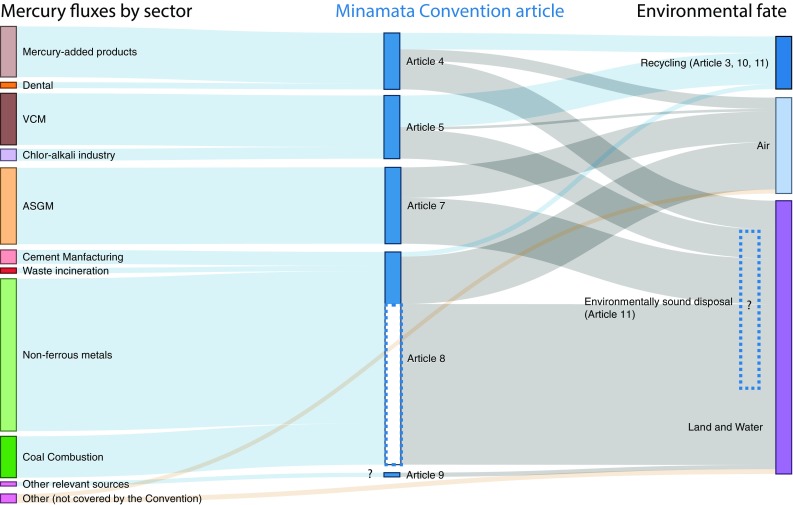
Global Hg sources and sinks for uses, emissions, and releases. The left side of the figure identifies the main sectors of commercial activity that either use Hg directly or emit or release Hg as a by-product. Their relative importance is indicated by the size of the colored bar. In the center, fluxes addressed by Articles under the Minamata Convention are indicated by blue bars. Unknown quantities (other relevant sources under Article 9, environmentally sound disposal) are indicated by question marks. The dotted line labeled Article 8 represents releases to land and water from sources mentioned in Article 8, as discussed in the text. The right side of the figure shows how much Hg from each sector goes to recycling or is emitted to air or released to land and water. Given that contaminated sites are created through releases to land, we have omitted them in Fig. 2 to avoid double-counting. Data used in the figure are from Horowitz et al. (2014), Streets et al. (2017a), United Nations Environment Programme (2017c), and estimates are discussed in the text

The mining of Hg, the intentional use of Hg in commercial products, industrial processes, and ASGM, and the presence of Hg as a by-product of combustion and other operations, have dispersed Hg widely into the environment (Obrist et al. 2018). Figure 2 synthesizes the available information on global-scale sources and sinks of Hg, organized by Minamata Convention Article. Many of these estimates have large uncertainty ranges, which are discussed further below.

Related to Article 3 on supply, primary Hg mining historically is the single largest source of mercury discharged to the environment, estimated to account for 27% of all-time cumulative Hg emissions and releases (Streets et al. 2017a). Despite long-standing collaborative international and national efforts to phase out Hg mining, there is evidence of a recent resurgence of informal mining in some countries such as Indonesia and Mexico (Camacho et al. 2016; Spiegel et al. 2018). Most of the resulting Hg supply is being used in the ASGM sector, either domestically or exported to other countries. Hg is also supplied to markets through recovery of by-product Hg from other non-ferrous mining activities as well as oil and gas processing, the closure or conversion of chlor-alkali facilities, the recycling of Hg-added products and Hg-containing wastes, and net changes in government or private sector stocks of Hg (United Nations Environment Programme 2017c).

Several studies have quantified total Hg emissions to air (Kim et al. 2010; Pacyna et al. 2010; Pirrone et al. 2010; United Nations Environment Programme 2013b; Zhang et al. 2015), and a few studies have quantified Hg releases to land and water (United Nations Environment Programme 2013b; Liu et al. 2016; Kocman et al. 2017). One study estimated that cumulative, all-time total Hg releases to land and water are 2.3 times the emissions of Hg to air (Streets et al. 2017a). Some case studies addressed integrated Hg flows in several countries (Chakraborty et al. 2013; Hui et al. 2016). Hg emission inventories are thought to be relatively accurate for some sources such as energy and industrial sectors, but have large uncertainties for other sources such as ASGM (Pacyna et al. 2016). Countries can use the UNEP Mercury Inventory Toolkit to help establish a national inventory of Hg uses, emissions, and releases.

As shown in Fig. 2, annual quantities of Hg use in products covered by Article 4 are estimated at 1200 Mg with an uncertainty range of 860–1540 Mg (United Nations Environment Programme 2017c, Table 23). It is estimated that 20% is emitted to air, 51% is released to land and water, and 29% is recycled (Horowitz et al. 2014 and personal communication). Hg inputs to processes covered by Article 5 are estimated for the VCM industry (1230 Mg, uncertainty range 1210–1240 Mg) and the chlor-alkali industry (280 Mg, uncertainty range 230–320 Mg) (United Nations Environment Programme 2017c). Emissions, releases, and recycling fractions from such processes are estimated as 4%, 46%, and 50%, respectively (Horowitz et al. 2014 and personal communication). Emissions and releases of Hg from ASGM covered by Article 7 are estimated at 1800 Mg (Streets et al. 2017a) with an the uncertainty range of 870–2600 Mg. About 45% of this Hg is emitted to air, and the rest to land and water. Estimated air emissions (97 Mg) and land and water releases (120 Mg) from sources not covered by the Minamata Convention include coal from residential use (Streets et al. 2017b) and transportation, iron and steel, and oil combustion sources (Streets et al. 2017a).

Streets et al. (2017a) estimated that 23% of Hg from the five sources of air emissions covered by Article 8 is emitted to the atmosphere. The same inventory estimated a large fraction of Hg releases from non-ferrous metals production, representing the difference between the Hg content in the processed ore and the calculated emissions to air, but the environmental fate of this Hg is not well known. The unshaded portion of the blue bar for Article 8 in Fig. 2 denotes the uncertainty regarding how such Hg releases may be addressed under the Minamata Convention, given that Article 8 only requires that BAT/BEP take into account the need to minimize cross-media effects. Only a small fraction of Hg from non-ferrous metal consumption is estimated to be recycled; the majority remains in wastes, which can be released to the environment (Wu et al. 2016). For Article 9 releases, the question mark illustrates the unknown number and size of Hg additional point sources not addressed in other provisions (i.e., sectors that result in releases to land and water other than those listed in Fig. 2). Each party must identify such domestic sources and report on control measures.

With respect to Articles 10 and 11 on storage and waste, data on the quantities of Hg are unavailable. In Fig. 2, these Articles are listed as relevant to recycling, along with Article 3 (Supply and Trade), as any Hg not reused will become waste. For Article 12 on contaminated sites, global inventories of sources and sinks, including those shown in Fig. 2, have historically omitted emissions and releases from Hg-contaminated sites. Such contamination is the result of different activities, including Hg mining and smelting, ASGM, large-scale precious metal processing, non-ferrous metal production, and major industrial uses in the chlor-alkali industry and other sectors. One study estimated that more than 3000 Hg-contaminated sites in different parts of the world release approximately 137–260 metric tons of Hg annually to the atmosphere and the hydrosphere (Kocman et al. 2013). Even though contaminated sites only make up a small percentage of total atmospheric emissions based on existing data, they are important Hg sources, especially for local water pollution, with many sites located in coastal areas where they release Hg into rivers, estuaries, and oceans (Randall and Chattopadhyay 2013).

To reduce intentional Hg uses, research and development have resulted in the introduction of Hg-free alternatives that are available on domestic and international markets for almost all of the products and processes that are covered by the Minamata Convention. Current focus is on accelerating the substitution to Hg-free products and processes, but this can be a time-consuming process in many markets, for both consumer goods and large-scale industrial processes like mercury-cell chlor-alkali plants that require substantial investment for conversion. On the topic of ASGM, one area of research is focused on refining and disseminating better and cheaper technologies for using Hg to separate the gold from the ore that will better protect the health of users and help to reduce emissions and releases into the environment (Sippl and Selin 2012). A related area of research focuses on socio-economic factors and strategies for changing the attitudes and behavior of people who live and work in ASGM mining communities towards reducing Hg use (Spiegel 2009; Saldarriaga-Isaza et al. 2015).

As countries phase out and ban commercial Hg uses, it will be necessary to deal with the excess Hg supply as well as discarded goods that contain Hg and Hg wastes. Regional assessments for Asia, Europe, Central America, and Latin America and the Caribbean projected that between the years 2010 and 2050 total excess Hg supply may exceed 28 000–46 000 tons (Maxson 2009; United Nations Environment Programme 2014; European Union 2015). This creates major institutional and technical challenges for environmentally safe storage and disposal of Hg. The COP will need to set threshold values for Hg content for the classification of Hg waste and provide guidance for the handling of such wastes. The Minamata Convention directs the COP to take into account technical guidelines for the identification and environmentally safe handling and disposal of Hg wastes that many Convention parties have already helped to develop under the global Basel Convention on the Control of Transboundary Movements of Hazardous Wastes and Their Disposal.

Research and development have also produced different kinds of technologies for controlling Hg emissions and releases, and several studies have reported on the degrees of effectiveness of these options (Krishnakumar et al. 2012; Sloss 2012; Trovant 2013; Ancora et al. 2016; Hu and Cheng 2016). Importantly, parties formulate their own national BAT and BEP standards (or ELV-equivalents) for controlling Hg emissions from new stationary sources in categories named in the Minamata Convention, based on a combination of domestic technical and economic factors (Lin et al. 2017). They shall also develop such national standards or other measures for controlling Hg emissions from existing sources as well as Hg releases. Because they are based on domestic factors, control standards may vary across regions and countries, but there is a general expectation that they should be strengthened over time. Countries may also apply different metrics when formulating their control standards. For example, the United States uses performance metrics (lb/Btu), while others including China and the European Union apply concentration limits (μg/m^3^).

#### Areas of further research needs

Large uncertainties in the flux estimates in Fig. 2, as described above, show the difficulty in quantifying and tracking both the global supply and use of mercury as well as its emissions and releases. Because several of these fluxes are uncertain, there is a lack of detailed information on trends, making it very difficult to quantify the relative impacts of control measures to address different aspects of the Hg life-cycle. To address the large uncertainties in global-scale Hg emissions and releases, there is a need for further systematic and harmonized data collection, model refinement, and analysis. There is often a lack of knowledge about local situations, especially for developing countries due to data shortages and the high cost of sampling and analysis.

Scientists can continue to play a critical role to ensure scientific credibility by generating additional data and new methods for emissions inventories and control technology effectiveness, as well as helping to develop alternative products and processes. They can also help improve the policy salience of this new scientific information by working with policy-makers to provide the most relevant information that is necessary to prioritize Hg sources and/or identify and evaluate the strengths and weaknesses of different kinds of policy options. Further, the political legitimacy of new scientific information can be enhanced if policy-makers believe that scientific experts have accounted for domestic conditions and relevant stakeholder input when helping to develop policy guidance (for example, for setting BAT, BEP, and ELV standards). To facilitate this work, scientists can take advantage of institutional mechanisms such as Minamata Convention expert groups, the Global Mercury Partnerships, and domestic stakeholder engagement processes.

Although there has been a decline in global primary Hg mining over the past few decades and it is believed that Hg currently is mined in only four countries—China, Mexico, Indonesia, and Kyrgyzstan—the recent reopening of old mines in Mexico and Indonesia is worrying from both policy and human health and environmental perspectives. There is a need for experts to work with government officials and representatives of international and civil society organizations to meet the Minamata Convention’s provisions on phasing out all Hg mining. Experts can also provide guidance to efforts that aim to reduce the supply to markets of Hg from the mining of other non-ferrous ores, oil and gas extraction, the decommissioning of chlor-alkali facilities, recycled Hg, and existing Hg stocks. There is also a need to collect and compile better export and import data in support of the Convention’s trade provisions (United Nations Environment Programme 2017c).

To improve Hg inventories, as well as enhance the ability to evaluate control options, several lines of additional research and measurements are needed. More cost-effective sampling and analytical methods would enable additional measurements, which are needed to support the deployment of air pollution control devices. Continuous measurements of Hg transformation and speciation under different operational conditions would enable better assessments of uncertainty and variability in inventories, as would more measurements of Hg emission from sources with large fluctuations (for example, waste incinerators and crematories). New measurements could facilitate the identification of local factors impacting Hg emissions to air, as existing inventories often apply these factors from other locations and regions, which may not reflect local conditions. Additional measurements of Hg releases to land and water from various sources of Hg in contaminated sites, as well as better methods for identifying and assessing risks, would also improve the ability to manage these sources.

Research is also needed to track Hg material flows, and monitor how emissions and releases change over time. Currently, most studies do not account for Hg demand using a supply chain perspective, which can provide more information on the drivers of uses, emissions, and releases, and help identify activities that are interconnected through waste and by-product flows (Hui et al. 2016; Wu et al. 2016). Developing a robust, detailed, bottom-up inventory that includes all relevant sources requires establishing a comprehensive national system for Hg material flows to track the Hg supply and trade, in and across societies, and movement in air, water, and soil. In many countries, this is challenging due to both data and resource shortages. However, further studies can help guide policy decisions to identify critical Hg use, emission, and release sources, and avoid secondary atmospheric Hg emissions.

Although the Minamata Convention’s measures on supply, uses, emissions, and releases under each Article are different, it is important to recognize that domestic efforts to comply with the provisions under one Article may lead to a decrease or increase of Hg emissions and releases that are addressed by another Article. For example, actions to curb Hg emissions from stationary sources under one Article may result in the capturing and storage of more Hg in the form of solid waste such as fly ash or gypsum from flue gas desulfurization, which is covered by another Article on waste management. If these solid wastes are allowed to be reused and heated to high temperatures, for example in cement production, the previously captured Hg may still be emitted to the air (Wang et al. 2014). As such, it is critical that estimates of emissions and releases, and policy decisions to control them and other parts of the Hg problem, consider the entire Hg life-cycle (Lin et al. 2017).

To put new knowledge into practice, scientists and engineers can engage in plans and policies for the deployment of technologies to control Hg emissions from major sources and support the design of national emission reduction plans. For example, a group of technical experts brought together by UNEP drafted guidance on BAT and BEP to assist parties in fulfilling their obligations under the Minamata Convention (United Nations Environment Programme 2015). Parties are likely to call on experts to provide similar advice on BAT and BEP during implementation at the national level. Where countries apply ELVs to domestic sources, these ELVs must reflect equivalent reduction levels that can be achieved using BAT. Therefore, policy-makers and regulators will need to rely on domestic experts as well as expert groups set up under the Convention to review and revise technical standards related to ELVs to ensure that they are consistent with progress on BAT. Scientists can also serve as experts in the design of other kinds of emissions reductions policies, especially for existing sources.

Research can assist parties to better understand the cost-effectiveness of various Hg control measures, which is challenging due to the limited information on current costs and difficulties in predicting cost trends and technical innovations. Future costs of Hg control measures could decrease due to economies of scale, commercial maturity, or technical innovation. Also, because different air pollution control devices result in different speciation profiles for the emitted and captured Hg, the choice of specific devices influences not only the absolute amount of Hg emitted, but also its transport and spatial deposition, which in turn determines who will experience the benefits of policy actions (Giang et al. 2015). To abate releases of Hg to water and land, scientists and engineers can help parties to identify domestic sources of such releases as well as to develop control measures and standards for these sources. Scientists can also work with public officials and local civil society and miners’ groups to find more efficient and cheaper technical methods, as well as promote behavioral changes, to reduce Hg emissions and releases from ASGM-related activities.

Researchers can continue to support public and private sector actors in developing and refining new Hg-free manufacturing processes and products that will support phase outs and restrictions on Hg use in products and processes in different parts of the world. Countries and researchers can draw on the extensive existing technical knowledge on substitutes and reduction measures, including those emerging from collaborative initiatives that were carried out under the Global Mercury Partnership (Sun 2017). Much focus is on phasing out the use of Hg catalysts in the VCM industry, which accounts for the largest use of Hg in China. Some policy recommendations suggest enhanced reporting and establishment of closed-loop systems, but the ultimate solution is to completely eliminate the need for Hg in the VCM industry in all countries (Wang et al. 2016). Similarly, while some short-term efforts to address Hg use in ASGM have focused on new technology development and deployment, researchers are also looking at longer-term approaches to phase out Hg use from this sector as well (Saldarriaga-Isaza et al. 2015).

It is important to expand knowledge and means for environmentally safe handling of Hg wastes as well as to further develop and disseminate guidelines and methods for easier and more cost-effective remediation practices for contaminated sites (Wang et al. 2012; Randall and Chattopadhyay 2013; Xu et al. 2015). North America and Europe continue to struggle to address older contaminated sites, and the number and severity of Hg-contaminated sites are increasing in Asia and other parts of the world (Li et al. 2009; Kocman et al. 2013). Engineers and scientists can play important roles in the further development and testing of different in situ and ex situ remediation options (Wang et al. 2012; Randall and Chattopadhyay 2013; Xu et al. 2015). The choice of a specific remedial approach should focus on site-specific parameters, as local conditions can vary tremendously across different sites (Randall and Chattopadhyay 2013; Xu et al. 2015). In response to the Minamata Convention requiring the development of guidance on managing contaminated sites, the COP at its first meeting called on governments and observers to nominate experts to draft such guidance (Decision MC-1/20).

### Support, awareness raising, and education

This area includes some of the enabling provisions of the Minamata Convention. The implementation of these Articles is important for providing a more robust international and national basis for successful Hg abatement.

#### Synthesis of existing knowledge base

Experience from other environmental treaties demonstrates that efforts to build management capacity benefit from concerted efforts across global, regional, national, and local governance scales (Selin 2010). The Minamata Convention Secretariat, like other treaty secretariats, plays an important global role in collecting, publishing, and disseminating data (Jinnah 2014). The Secretariat works alongside major intergovernmental organizations, including UNEP, the World Health Organization (WHO), the United Nations Development Programme, the United Nations Institute for Training and Research, and the United Nations Industrial Development Organization, in hosting and supporting capacity-building programs and training sessions that promote information exchange, including helping countries to prepare for treaty ratification and implementation. The Regional Centers for the Basel Convention and the Stockholm Convention on Persistent Organic Pollutants, which are working with countries on capacity-building and technology transfer issues for those agreements, have also begun to assist the parties to the Minamata Convention (Selin 2012; United Nations Environment Programme 2016).

Studies and field experiences show that the effective design and implementation of awareness-raising and science-based education programs require a comprehensive and long-term approach. Programs must be flexible enough so that they can be adjusted over time and be tailored to specific local legal, political, economic, social, cultural, and environmental contexts (Chouinard and Veiga 2008; Sousa and Veiga 2009; United Nations Development Programme 2009; Arctic Monitoring and Assessment Programme 2015). Efforts to disseminate information and new methods and technology to change the behavior of targeted groups must include close and repeated interactions between authorities, experts, and community members (García et al. 2015). Many of the more effective educational approaches also target key individuals whose actions will influence the decisions made by others, and take into account outcomes of community-wide efforts to change attitudes and behaviors (Sippl and Selin 2012).

The use of scientific and technical knowledge is key to efforts to change attitudes and behavior among particularly vulnerable populations, and this is especially true in the ASGM sector. Studies show that programs aiming to reduce Hg use and exposure should include both the dissemination of science-based information on the environmental and health risks from Hg as well as the engagement with miners to develop and apply new technologies for reducing Hg emissions and exposure (Zolnikov 2012). Further, encouraging miners to shift to Hg-free techniques will require more than a demonstration of alternative technologies—it will require an understanding of local socio-economic and cultural factors and relationships among different actors along the gold supply chain (Spiegel et al. 2018). In addition, a large proportion of ASGM takes place in the informal sector, creating a host of legal, political, and management challenges and land-use conflicts that must be considered (Hilson and Gatsinzi 2014).

Dentistry is another major area of intentional Hg use where efforts are underway to raise awareness, and to generate and communicate science-based information to achieve behavioral change (Mackey et al. 2014). For example, a civil society-initiated campaign to phase down the use of Hg amalgams in Asia and Africa faced initial resistance from policy-makers and dentists who did not believe that Hg posed a risk. To overcome this skepticism, scientists from countries in both regions used a portable device to measure Hg levels in the air in dental offices (Ali and Khawja 2015). On-the-spot measurements demonstrating high Hg levels proved to be a strong and salient method of risk communication with dentists across different cultural settings, and helped garner support for changing workplace practices to reduce Hg exposure. The credibility of the education campaign was enhanced by a WHO publication and other studies on options for Hg-free dentistry (World Health Organization 2010; Ferracane 2011).

Many public health education campaigns focus on the presence of methylmercury (MeHg) in fish and seek to raise awareness and communicate appropriate dietary guidelines, especially for vulnerable populations such as pregnant women and small children (Mergler et al. 2007; Mahaffey et al. 2011). Studies show that in developing science-based dietary guidelines, it is critical that experts work closely with local communities, including indigenous communities where the harvesting and consumption of seafood are integral to long-standing cultural values and practices (Arctic Monitoring and Assessment Programme 2015). It is important that nutritional benefits of fish consumption are evaluated against risks of Hg exposure when designing consumption guidelines (Mahaffey et al. 2011). Some newer diet-related research has also focused on Hg in rice, which is sometimes grown in Hg-contaminated paddy fields (Li et al. 2009; Rothenberg et al. 2014; Hsu-Kim et al. 2018). This research suggests that new dietary guidelines around foods other than fish may need to be developed for vulnerable populations.

#### Areas of further research needs

The ability of parties to meet Minamata Convention goals depends on the use of scientifically credible, policy salient, and politically legitimate information that recognizes the political, economic, social, and cultural dimensions of collective and individual actions required to reduce Hg exposures and risks. Improving programs for building capacity and raising awareness to change human behavior requires research on the design of effective communication strategies and programs within different knowledge systems. Specialists can use their expertise to develop and deploy effective communication tools and craft messages that are tailored to local communities, policy-makers, and the general public (Arctic Monitoring and Assessment Programme 2015). Efforts to design better science-based communication strategies in turn are dependent on improved quality and quantity of data on Hg pollution, including biomonitoring data for different species and ecosystems as well as data on the health effects of low-level Hg exposure to different human populations (Arctic Monitoring and Assessment Programme 2015).

Many collaborative efforts on capacity-building, awareness-raising, and implementing science-based education programs continue to focus on the ASGM sector, as past initiatives across South America, Africa, and Asia have not been sufficient to address problems of Hg use, emissions, and releases that negatively impact the environment and human health. To enhance the political legitimacy of their work, researchers can engage national governments, local stakeholder groups, and international organizations that collaborate around the development of ASGM National Action Plans under the Minamata Convention. These plans must include technical approaches to mercury reduction, including introduction of mercury-free mining techniques, but also must contain strategies to address the widespread phenomenon of ASGM miners working outside of national laws without formal mining rights. The informality and/or illegality of miners often causes conflicts with both authorities and large mining corporations (Sippl and Selin 2012) and undermines the ability of miners to acquire financing needed to purchase better technologies. Addressing these types of complex legal and political issues requires the establishment of greater trust between technical experts, authorities, miners, and other community members (Spiegel et al. 2018).

To minimize environmental and health impacts to ASGM miners and their communities, including urban processing centers, experts can help to refine existing technologies and/or develop new mining methods, and can work with individual miners to introduce and use them. Importantly, efforts to expand the introduction of Hg-free techniques in ASGM communities must consider the particular local social and economic drivers of the use of Hg, in order to see more widespread uptake of new methods and technologies. It is also necessary to further develop and apply performance indicators to evaluate education and technology diffusion programs (Sousa and Veiga 2009) to measure the speed and effectiveness of uptake. Researchers can also help design and evaluate programs that expand opportunities for alternative livelihoods for miners, and that facilitate collaboration among mining communities, local and national governments, and international organizations in support of sustainable development overall.

Further communications research can support efforts to phase out the use of Hg and Hg-containing products, by considering both scientific information and local conditions and perceptions that underpin these uses. For example, this could mean working with dental professionals and patients to accept alternatives to Hg amalgam, as well as assuring medical practitioners about the efficacy of Hg-free alternatives to existing Hg thermometers and sphygmomanometers. Educational campaigns in Africa discovered that women still use Hg-containing skin lightening products despite the health risks because women with fair skin are perceived to be more attractive by prevailing social standards (Agorku et al. 2016). Because these social pressures have not been adequately addressed, legislation in several countries against these products has been difficult to implement. In addition, there is a continuing need for localized research to examine health risks from dietary intake of Hg-containing food, including fish and rice, and to devise appropriate consumption guidelines for different communities (Meng et al. 2014; Arctic Monitoring and Assessment Programme 2015).

### Impacts and effectiveness

This area includes another set of enabling provisions under the Minamata Convention. The effective use of these Articles is critical to the ability to meaningfully evaluate the effectiveness of the Convention, and to identify priority areas for greater Hg abatement efforts.

#### Synthesis of existing knowledge base

Scientific research has addressed the Hg life-cycle in ways that can assist policy-makers and other stakeholders in tracing the causal chain from Hg policies to health and environmental impacts. Changes in anthropogenic Hg emissions may result from implementation of Convention provisions, from other socio-economic or environmental policies, or both. Changes in emissions in turn result in changes in Hg deposition to ecosystems, and subsequent conversion to MeHg (Hsu-Kim et al. 2018; Obrist et al. 2018). Finally, changes in human and environmental exposure and adverse impacts result from different forms and levels of Hg exposure. Yet, efforts to identify policy signals among these impacts must also account for factors other than the Convention that might affect outcomes.

Much analysis to date has been aimed at reducing uncertainties in factors that affect the first part of the causal chain from policies to impacts, involving Hg emissions, cycling, and environmental behavior. In addition to uncertainties due to incomplete scientific data and understanding, some of these factors are highly variable and source- and location-dependent. For example, the processes that drive rates of atmospheric depletion and deposition, such as Hg oxidation (Ariya et al. 2015) and meteorological factors, vary over spatial and temporal scales. Once Hg is deposited on land or in water, local conditions such as temperature and the amounts of oxygen, organic matter, and sulfate drive the transformation of elemental Hg into more toxic MeHg (Faganeli et al. 2014; Wentz et al. 2014; Gascon Diez et al. 2016). Hg exposure levels as well as health outcomes vary among populations due to differences in susceptibility to Hg impacts. These impacts can also change over time due to factors, such as climate change, not related to Hg policy (Eagles-Smith et al. 2018).

Measuring and monitoring Hg levels and trends in the environment is a key input to the policy to impact causal chain analysis. Monitoring provides data on mercury in the environment, can identify significantly impacted ecosystems and human populations, and forms a basis for testing and calibrating models. For example, monitoring near specific emission sources can detect changes in Hg deposition over relatively short time scales (e.g., Lindberg et al. 2007). Such local- to regional-scale observations can be critical for demonstrating progress that can be reasonably attributed to local source reductions. In contrast, the response of global atmospheric Hg concentrations to Minamata Convention provisions will be complex and influenced by a wide range of environmental and policy factors. In fact, global Hg deposition may increase in the short term even under some emissions reduction scenarios, as mobilization of legacy Hg exceeds sequestration in the environment (Sunderland and Selin 2013). Because of these complexities, interpretation of monitoring data can be challenging.

Existing atmospheric monitoring data show mixed trends. Both decreasing (Zhang et al. 2016) and increasing (Martin et al. 2017) trends have been attributed to changes in emissions, while other studies have focused on the influence of the ocean (Chen et al. 2015). Trends in wet deposition are even more variable. Studies at sites in North America show a combination of increases and decreases that are sensitive to location and the time period of analysis (Weiss-Penzias et al. 2016). For fish concentrations, decreases in the Atlantic (Lee et al. 2016) and increases in the Pacific (Drevnick et al. 2015; Drevnick and Brooks 2017) were both linked to global-scale emissions changes. Further, Hg levels in open ocean fish will likely begin to decrease within years to decades as a result of reduction measures while Hg in fish from coastal areas contaminated by legacy Hg may take many decades, or even centuries, to decline, due to differences in Hg cycling in these different ecosystems (Chen et al. 2016). Environmental processes for Hg are also impacted by other global changes, including climate change (Krabbenhoft and Sunderland 2013).

To complete the causal chain analysis, some research uses information on Hg emissions, cycling, transport, and deposition to simulate the health and economic impacts resulting from anticipated policy choices under the Minamata Convention. An economic evaluation of the health benefits of advanced Hg emission controls in China projected that cumulative Hg-related health benefits could exceed $400 billion by 2030 (Zhang et al. 2017). These kinds of analyses also make it possible to quantify the relative importance of various sources of uncertainty and variability across the chain of policies-to-impacts when estimating human health and economic impacts of the Convention. Uncertainties in the Hg cycling and ecosystem dynamics that influence the timescale of changes in Hg concentrations have been found to strongly affect benefit estimates (mainly because of time discounting of future benefits) (Giang and Selin 2016).

#### Areas of further research needs

Evaluating whether the Minamata Convention is protecting human health and the environment from Hg ultimately requires mobilizing policy-relevant scientific knowledge across the complex chain of causality and attribution from policies to impacts. However, initial effectiveness evaluations are likely to be informed primarily by data and models representing early and intermediate steps in this causal chain. Here, the COP may use an approach similar to the framework for effectiveness evaluation used by the Stockholm Convention. This includes developing outcome indicators to reflect changes in impacts on human health and the environment (United Nations Environment Programme 2017a), complemented by process indicators that indicate levels of compliance with control measures and other mandates (United Nations Environment Programme 2013a). In doing so, it is important that the data gathering and evaluation process helps to ensure not only the scientific credibility but also the policy salience and political legitimacy of the data and the effectiveness evaluation reports.

The use and interpretation of outcome indicators to measure effectiveness pose an ongoing scientific challenge. Despite extensive efforts to understand the Hg cycle, scientific uncertainties, and environmental variabilities limit the ability to link global changes in Hg emissions to environmental concentrations and exposures, and obscure the ability to attribute these changes to Minamata Convention-related implementation measures (Selin 2014b; Kwon and Selin 2016; Obrist et al. 2018). To address these uncertainties, there is a need to collect more empirical data on Hg emissions and concentrations over longer time periods and geographical areas, on environmental factors that affect atmospheric transport and biogeochemical cycling, and on the factors that drive changes in Hg bioaccumulation, biomagification, exposure, and toxicity. In addition, the development of a reliable baseline is critical for evaluating the impacts and effectiveness of Convention provisions. While data are available in many cases for Hg emissions and releases (e.g., from the Global Mercury Assessment), data may be lacking for other important variables such as environmental concentrations in particular species or regions. Further, political considerations may complicate which information the COP ultimately chooses as its baseline.

To address the numerous analytical challenges in monitoring Hg, scientists need to develop more cost-effective monitoring designs, as well as new sampling and analytical methods, including methods for quality control of measurements, particularly for oxidized and particulate Hg (Jaffe et al. 2014). Approaches to collect and analyze monitoring data also need to be harmonized (Bank et al. 2014). Experts may recommend concentrating on more comparable, reliable, and longer-term monitoring in fewer locations, and place greater emphasis on strategic measurements in key human populations and ecosystems, rather than pursuing a greater number of measurements (Gustin et al. 2016). Many existing monitoring networks need to be expanded and enhanced to contribute the data needed to answer important policy questions. One such effort concerns the Asia–Pacific Mercury Monitoring Network, which aims to create a coordinated Asia-wide network to monitor Hg transport and deposition. Scientists can guide such efforts, including how to appropriately interpret monitoring data given different spatial and temporal scales of Hg processes in the environment and the non-linear relationship of Hg deposition and biotic MeHg concentrations.

Researchers will need to improve models to make use of such data in more robust ways. It is especially critical to develop a better understanding of the behavior of Hg in conditions common to tropical regions, where a large proportion of global Hg emissions now occur (United Nations Environment Programme 2013b). New methods are needed for integrating this scientific information with other social and economic information into a coherent framework against which the effectiveness of the Minamata Convention can be assessed. In response to Decision MC 1/9 taken at its first meeting, the COP has established an ad hoc group of experts and observers to help design such a framework, providing a key point of intervention for scientists and researchers to directly affect the methods and data sources that are chosen for future effectiveness evaluations.

The process indicators that are developed to complement outcome indicators will need to rely on reporting by the parties on the domestic implementation of specific control measures, but can be supplemented by targeted data collection on specific expected policy outcomes, such as the declining market availability of Hg-containing products to be phased out by 2020. Additional research can build on existing proposals for a suite of such process indicators intended to reflect the effectiveness of key control provisions related to trade, products and processes, ASGM, and air emissions, in addition to recommending longer-term indicators based on biomonitoring of ecosystems and human populations (Evers et al. 2016). Specific process indicators can be proposed for the short term (less than 6 years), medium term (6–12 years), and long term (greater than 12 years), to match different target dates for the implementation of Minamata Convention provisions.

Using a broad definition of effectiveness, research can help strengthen the evaluation of Minamata Convention provisions by developing metrics related to changes in underlying social drivers that influence the uses and emissions of Hg. For example, indicators can be developed that reflect the increased capacity of scientists, governments, and others to manage Hg according to Articles 14 and 17. Effectiveness of efforts under Article 18, to raise public awareness of Hg, can be evaluated by how well they have been able to modify cultural and social views among critical target populations whose behavior influences the rate of change of Hg use, emissions, and releases. Such changes are hard to quantify, much less directly observe and measure, and will require collaboration between social scientists, educators, communication specialists, and program evaluation specialists to develop appropriate indicators (Macdonald et al. 1996; Centers for Disease Control 1999; Abroms and Maibach 2008).

## The future of Hg science and governance

The Minamata Convention is set to play a central role in environmental and human health protection from Hg-related exposures and risks. The most recent effectiveness evaluation of the Stockholm Convention notes that an environmental treaty can act as an important catalyst for expanded research, monitoring, and modeling and for bringing together findings from different parts of the world (United Nations Environment Programme 2017b). Future Hg research can support Convention implementation efforts in numerous ways and feed into policy-making and management at multiple times and entry points. This paper should not be seen as an exhaustive summary of all the areas in which the larger research community can contribute, but rather as an effort to connect scientifically credible research with key Convention implementation needs, and also discuss ways in which it is possible to enhance its policy relevance and political legitimacy. In this regard, there is a high demand for interdisciplinary expertise and perspectives.

Scientists who want to assist international, national, and local efforts on Hg abatement can do so through multiple institutional mechanisms. Internationally, scientists can contribute to scientific assessments, either as participants or reviewers. They can assist or participate in the work of Minamata Convention bodies and expert groups, such as the ones that recommend BAT and BEP standards or propose guidelines for the effectiveness evaluation. Scientists can contribute to international projects that support countries in taking legal, political, regulatory, and administrative measures toward Convention implementation. Scientists can inform, or even participate alongside, government officials who are members of national delegations to meetings under the Convention. They can also engage with policy-makers who set regulatory standards and public officials who work on Hg abatement, including participating in public, multi-stakeholder processes for the development and implementation of ASGM National Action Plans. Scientists can help develop better pollution prevention technologies or cheaper Hg-free production processes and Hg-free commercial products. These types of efforts may involve collaboration between universities, the private sector, and governments.

As the parties move forward with implementation of the Minamata Convention, building on work carried out under the Global Mercury Partnership and other international programs and legally binding agreements (Selin and VanDeveer 2004), there is a need to strengthen multilevel approaches to Hg management across global, regional, national, and local governance scales (Selin 2014a). Governance-focused studies can continue to analyze how activities and decisions in different fora and governance levels by international organizations, countries, civil society organizations, and scientists are linked, and explore ways in which such linkages can be used to create synergistic effects toward better Hg management. Such studies should be carried out alongside more research into the impacts of Hg on human health and the environment and how Hg cycles through different environmental media, since better Hg management is dependent on a combination of scientific information from different sources, fields, and disciplines.

To enable different forms of continued monitoring and applied Hg-related research in support of policy-making and treaty implementation, it is critical to build and support basic scientific and analytical capabilities, especially in developing regions of the world where the lack of such capabilities is a central issue. Developing and newly industrialized countries are likely to be the ones where the most wide-ranging policy measures are needed, as these countries now represent the majority of global Hg supply, uses, emissions, and releases. Such measures may include major cuts in atmospheric Hg emissions from coal-fired power plants and other major stationary sources, the phasing out of Hg use in products and processes, and the reduction and ultimately elimination of Hg use in ASGM. Building implementation capacities in these countries and supporting technology transfer will be critical to the success of the Minamata Convention.

An essential governance challenge is to simultaneously address Hg supply and demand in a coordinated way (Greer et al. 2006; United Nations Environment Programme 2017c), and this paper focuses mainly on scientific contributions to the implementation of existing treaty provisions that address currently prioritized supply and demand elements of the Hg life-cycle. However, as the COP oversees the implementation of the Minamata Convention, including the use of the facilitative compliance mechanism (Templeton and Kohler 2014), current mandates will be reviewed and new requirements can be added. For example, the COP may identify other sources of Hg emissions and releases, update BAT and BEP guidance, and/or introduce new provisions to account for cross-media Hg management. In addition, the COP may expand focus to additional Hg-containing products and processes. On all such decisions, the COP will benefit from existing and developing scientific and technical information.

The implementation of the Minamata Convention also intersects with other environmental treaties. As discussed earlier, greater attention to the environmentally safe handling and disposal of Hg wastes creates policy-making and management linkages with parallel efforts under the Basel Convention (Selin 2010), as Basel and Stockholm Convention Regional Centers assist countries also on Hg abatement. Climate change mitigation efforts under the Paris Agreement have implications for Hg pollution and its impacts (Arctic Monitoring and Assessment Programme 2015). For example, climate-induced changes in food web structures may enhance bioaccumulation and biomagnification of MeHg in some marine species (Jonsson et al. 2017; Obrist et al. 2018). Finally, Hg management takes place in a broader context of the global sustainable development agenda, linked to the UN Sustainable Development Goals for good health and well-being, clean water and sanitation, affordable and clean energy, responsible consumption and production, and sustainability of life below water.
